# Effects of Cigarette Smoking on Oxidative Stress, DNA Damage, Immunological Profile, Viral Susceptibility, and Survival in Patients with Chronic Obstructive Pulmonary Disease

**DOI:** 10.3390/biom16071009

**Published:** 2026-07-10

**Authors:** Antonio de Iure, Laura Vitiello, Stefania Proietti, Paola Fortugno, Dolores Limongi, Carla Prezioso, Fabrizio Maggi, Guido Antonelli, Barbara Picconi, Carlo Tomino, Giorgio Felzani, Stefano Bonassi, Patrizia Russo

**Affiliations:** 1 Department of Human Sciences and Promotion of the Quality of Life, San Raffaele University, Via di Val Cannuta 247, 00166 Rome, Italy; antonio.deiure@uniroma5.it (A.d.I.); paola.fortugno@uniroma5.it (P.F.); dolores.limongi@sanraffaele.it (D.L.); carla.prezioso@uniroma5.it (C.P.); barbara.picconi@uniroma5.it (B.P.); stefano.bonassi@sanraffaele.it (S.B.); patrizia.russo@uniroma5.it (P.R.); 2 Laboratory of Experimental Neurophysiology, IRCCS San Raffaele, Via di Val Cannuta 247, 00166 Rome, Italy; 3 Clinical and Molecular Epidemiology, IRCCS San Raffaele, Via di Val Cannuta 247, 00166 Rome, Italy; 4 AgEA Coordinating Body, 00185 Rome, Italy; s.proietti@agea.gov.it; 5 Human Functional Genomics Laboratory, IRCCS San Raffaele, Via di Val Cannuta 247, 00166 Rome, Italy; 6 Laboratory of Microbiology, IRCCS San Raffaele, Via di Val Cannuta 247, 00166 Rome, Italy; 7 National Institute for Infectious Diseases “Lazzaro Spallanzani”—INMI IRCCS, Via Portuense 292, 00149 Rome, Italy; fabrizio.maggi@inmi.it; 8 Department of Molecular Medicine, Sapienza University, Viale Porta Tiburtina 28, 00185 Rome, Italy; guido.antonelli@uniroma1.it; 9 Microbiology and Virology Unit, Sapienza University Hospital Policlinico Umberto I, 00161 Rome, Italy; 10 Scientific Direction, IRCCS San Raffaele, Via di Val Cannuta 247, 00166 Rome, Italy; carlo.tomino@sanraffaele.it; 11 San Raffaele Sulmona, Viale dell’Agricoltura, 67039 Sulmona, Italy; giorgio.felzani@sanraffaele.it

**Keywords:** chronic obstructive pulmonary disease, cigarette smoking, oxidative stress, DNA damage, Torque teno virus, immune dysregulation, nicotinic receptors, survival, pulmonary rehabilitation, precision medicine

## Abstract

Background: Cigarette smoking promotes persistent systemic alterations in COPD, yet the interplay among genetic susceptibility, oxidative stress, immune dysregulation, impaired control of persistent viral replication, and long-term outcomes remains incompletely understood. Methods: We conducted an observational study in 102 patients aged ≥70 years with severe-to-very-severe COPD undergoing pulmonary rehabilitation. Current smokers (n = 38) were compared with never/former smokers (n = 64). Analyses included Chr15q25 genotyping (rs16969968), oxidative stress biomarkers (tail intensity, 8-OHdG, MDA, and bilirubin), hematological and immunological parameters, α7nAChR expression, TTV load as a surrogate marker of immune competence, latent virus prevalence, and five-year survival assessed by multivariable Cox regression. Results: Current smokers exhibited significantly higher DNA damage (tail intensity, *p* = 0.001; 8-OHdG, *p* = 0.002), lower bilirubin levels (*p* = 0.031), increased neutrophil and CD4^+^ T-cell counts (*p* = 0.031 and *p* = 0.028, respectively), altered α7nAChR expression on CD4^+^ T cells (*p* = 0.030), and higher TTV load (*p* = 0.002) than never/former smokers. The rs16969968 AA genotype was more frequent among current smokers. In survival analyses, an elevated WBC count was independently associated with increased mortality risk (HR 1.12, 95% CI 1.01–1.23; *p* = 0.035), whereas higher bilirubin levels showed a protective association. TTV load, smoking status, and FEV1 were not independently associated with mortality. Conclusions: In severe-to-very-severe COPD, smoking is associated with a distinct biological profile characterized by enhanced oxidative DNA damage, systemic inflammation, immune remodeling, reduced antioxidant defenses, and impaired control of persistent viral replication. WBC and bilirubin emerged as the biomarkers most consistently associated with long-term outcomes. These findings support integrated biological profiling as a tool for risk stratification and precision-guided rehabilitation in advanced COPD.

## 1. Introduction

Tobacco use remains one of the leading preventable causes of morbidity and mortality worldwide. Although its association with lung cancer and chronic obstructive pulmonary disease (COPD) has been firmly established for decades, smoking continues to drive a substantial burden of non-communicable diseases, including cardiovascular and cerebrovascular disorders [1,2]. Large-scale epidemiological studies have shown that cigarette smoking increases lung cancer risk by 10- to 30-fold and represents the principal modifiable risk factor for COPD [3].

COPD is characterized by persistent airflow limitation resulting from small airway remodeling and emphysematous destruction [2,3]. However, spirometric impairment alone fails to capture the full complexity of the disease, as COPD is highly heterogeneous and frequently accompanied by systemic manifestations and multimorbidity, including cardiovascular disease, anemia, metabolic alterations, and immune dysfunction [4]. These features underscore the need for multidimensional, mechanism-oriented approaches that extend beyond lung function assessment.

Oxidative stress represents a central pathogenic mechanism linking cigarette smoke exposure to the development and progression of COPD. Tobacco smoke contains abundant reactive oxygen and nitrogen species that induce DNA damage, lipid peroxidation, protein modification, and chronic inflammation [5]. Excessive reactive oxygen species (ROS) generation, together with impaired antioxidant defenses, promotes cellular senescence, defective autophagy, altered immune responses, and corticosteroid resistance, thereby contributing to disease persistence and progression [6].

Oxidative stress also plays a critical role in host–virus interactions. Redox imbalance impairs antiviral signaling, disrupts epithelial barrier integrity, and amplifies inflammatory responses, increasing susceptibility to respiratory viral infections and prolonging exacerbations in patients with COPD [7,8]. Cigarette smoke further compromises antiviral defenses by interfering with toll-like receptor signaling and facilitating viral entry into airway epithelial cells [9,10]. This bidirectional interaction between viral infections and COPD contributes to accelerated lung function decline and systemic complications.

In parallel, smoking exerts profound immunomodulatory effects, altering both innate and adaptive immunity. Dysregulation of CD4^+^ and CD8^+^ T cells, expansion of regulatory T-cell subsets, impaired macrophage and neutrophil function, and altered B-cell phenotypes have been consistently reported in smokers and patients with COPD [11]. These immune alterations may facilitate viral persistence and reactivation, particularly in individuals receiving corticosteroid therapy.

Despite substantial advances in tobacco control, COPD-related morbidity and mortality continue to rise globally. The Lancet Commission on COPD has highlighted the need to reconceptualize disease determinants by integrating tobacco exposure, host genetic susceptibility, early-life insults, recurrent pulmonary infections, and chronic environmental pollutants into a unified etiopathogenetic framework [12,13].

Within this framework, molecular and cellular biomarkers are critical for elucidating pathogenic mechanisms and enabling precision medicine in COPD. Key targets include: genetic predisposition, such as polymorphisms affecting nicotine dependence (rs16969968) [14]; oxidative stress markers, including ROS, 8-hydroxy-2′-deoxyguanosine (8-OHdG), and bilirubin levels [15,16,17]; genome instability [18]; programmed cell death pathways, such as apoptosis, necroptosis, and NETosis [19,20]; immune dysregulation, including altered T-cell subsets and cytokine profiles [21]; and susceptibility to microbial or viral reactivation [22]. Integrating these biomarkers with longitudinal clinical and environmental data may facilitate mechanistic endotyping, early disease detection, prognostic stratification, and targeted therapeutic interventions.

The present study aimed to investigate the impact of tobacco exposure on immune competence, susceptibility to viral replication, and long-term clinical outcomes in patients with advanced COPD undergoing pulmonary rehabilitation.

Specifically, the objectives were to:

- Evaluate the contribution of genetic susceptibility and smoking exposure by assessing the association between the rs16969968 polymorphism and smoking-related clinical characteristics.
- Characterize smoking-associated immune dysregulation through the analysis of peripheral T-cell populations (CD3^+^, CD4^+^, and CD8^+^ subsets) and α7 nicotinic acetylcholine receptor (α7nAChR) expression.
- Assess susceptibility to viral replication and immune competence using Torque teno virus (TTV) load as a surrogate marker of host immune surveillance, and examine its relationship with smoking status and immunological parameters.
- Investigate the prevalence of selected respiratory and systemic viral infections and explore their association with smoking exposure and immune status.
- Determine the prognostic impact of smoking burden, immune dysfunction, TTV load, and neutrophil extracellular trap formation (NETosis) on five-year survival.
- Explore the potential clinical utility of TTV quantification and smoking-related biomarkers for risk stratification and the development of personalized rehabilitation strategies in COPD.

Importantly, this study was not designed to quantify the entire spectrum of viral infections affecting COPD patients. Rather, TTV load was employed as a quantitative surrogate marker of immune competence and susceptibility to persistent viral replication, providing a dynamic and host-centered measure of immune surveillance.

## 2. Materials and Methods

### 2.1. Study Design and Participants

A prospective observational study was conducted involving 102 individuals aged 70 years or older, all diagnosed with advanced COPD and admitted to the Pulmonary Rehabilitation (PR) Unit of IRCCS San Raffaele in Rome between September 2013 and December 2015 to undergo a structured three-week PR program. Upon hospital admission, peripheral blood samples were collected and preserved at −80 °C. PBMCs were isolated using SepMate™ tubes (STEMCELL Technologies, Cambridge, MA, USA), which allow for consistent and straightforward isolation in a 15-min protocol. Centrifugation with SepMate™ was carried out for 10 min with the brake applied; subsequently, the isolated PBMCs were transferred into a fresh tube and washed [23]. The PBMCs were then resuspended in cryopreservation medium, gradually cooled to very low temperatures, and stored in liquid nitrogen (below −135 °C) until further use. Urine specimens were collected at admission into plastic containers that were subsequently frozen at −20 °C. Follow-up was carried out by tracing participants to their place of residence, and data on survival status and cause of death were retrieved from the relevant municipal records. The observation period extended to five years after discharge.

The study protocol received approval from the Ethics Committee of IRCCS San Raffaele Roma (Prot. 15/2013), and written informed consent was obtained from all participants at the time of enrollment. All patients were categorized into three groups based on smoking history, following the National Center for Health Statistics definitions: current smokers (≥100 cigarettes lifetime and still smoking), former smokers (≥100 cigarettes lifetime but quit at the time of assessment), and never-smokers (<100 cigarettes lifetime). Classification was further validated by urinary cotinine testing using the SureScreen Instant Cotinine Test (Sherwood Business Park, Little Oak Drive, NG15 0DR), certified under ISO 9001/13485 [24,25] and CE-marked (more than 99% accuracy in cotinine detection COT) with the cutting level of 100 ng/mL and the detection time of 1–7 days), which identifies recent nicotine exposure but does not provide information on the duration of smoking cessation.

All patients at admission received a European Union (EU) validated questionnaire to estimate food items intake [26]. Given the robust evidence demonstrating beneficial effects starting from a daily consumption of vegetables [27], we compared patients eating vegetables once a day or more frequently (higher intake) with those reporting a low/moderate intake of vegetables (from 4 times a week up to a minimum of less than once a week).

Nobody answered ‘never’ and 25 patients did not answer.

For hematological, DNA damage, oxidative, inflammatory, and immunological analyses, patients were stratified into two categories: (1) never and former smokers combined, and (2) current smokers only. This approach was adopted because former smokers had abstained for at least two years, were negative for cotinine testing, and their values did not significantly differ from those of never smokers (Supplementary Table S1).

### 2.2. Chr15q25 Genetic Variant (rs16969968)

Patients were genotyped for the rs16969968 variant (CHRNA5, NM_000745.4:c.1192G>A; p.Asp398Asn) using a TaqMan^®^ allelic discrimination assay (TaqMan^®^ assay ID C__26000428_20) according to the manufacturer’s instructions (Thermo Fisher Scientific, Waltham, MA, USA). Five to ten ng of DNA was amplified by PCR using TaqMan^®^ genotyping master mix and the QuantStudio1 real-time PCR platform (Thermo Fisher Scientific, Waltham, MA USA 02451). Allelic discrimination was performed using TaqMan Genotyper Software version 1.6 (Thermo Fisher Scientific). Genotype call rate was 77.5%: 19 patients were homozygous GG (WT; 24.1%), 43 were heterozygous AG (SNP carrier; 54.5%), and 17 were homozygous AA (SNP; 21.5%). Collectively, individuals carrying at least one mutant allele (AA + AG) accounted for 60 subjects (75.9%).

Patients were also genotyped using a PCR-RFLP assay. The target region was amplified from patients’ gDNA by PCR using TaKaRa LA Taq (TAKARA Bio Inc., Shiga, Japan) with primers: forward 5′-AGTCATGTAGACAGGTACTTCACTCAG-3′, reverse 5′-TGGAAGAAGATCTGCATTTG-3′. PCR products were subjected to Taq I restriction enzyme digestion, which selectively cleaves the wild-type allele. Digestion products were separated by agarose gel electrophoresis and imaged using a LAS-1000 Imager (Aïda software, Raytest, Courbevoie, France). The assay was performed as reported by Cherif et al. [28].

### 2.3. Blood Test

The number and percentage of peripheral blood cells were evaluated during routine laboratory analysis using standard blood count automated methods (Beckman Coulter LH500, Beckman Coulter, Milan, Italy).

### 2.4. Markers of Oxidative Stress

The following markers of oxidative stress were measured to evaluate differences between current and former/never smokers.

#### 2.4.1. Malondialdehyde (MDA) Assay

MDA quantification was determined as previously described [29].

#### 2.4.2. 8-Hydroxy-2′-deoxyguanosine (8-OHdG) Assay

8-OHdG was determined as previously described [29].

### 2.5. Alkaline Comet Assay

The complete detailed procedure for the assay can be found in our previous work [29]. In summary, DNA damage from lymphocytes was evaluated after lymphocyte lysis, DNA denaturation, electrophoresis on agarose gel, and staining, using the Comet Assay IV software version 4 (Instem, London, UK). Tail intensity values (TI, % DNA in comet tail) were calculated from 100 comets counted for each individual.

### 2.6. Markers of Inflammation

Interleukin-6 (IL-6) levels and C-reactive protein were determined as previously described [29].

### 2.7. Flow Cytometry Analysis

PBMCs stored in liquid nitrogen were thawed and resuspended in RPMI plus 10% FBS, 2 mM L-glutamine, 1% sodium pyruvate, 1% non-essential amino acids, and 1% penicillin/streptomycin (all from SIAL, Rome, Italy) at 1 × 10^6^ cells/mL. Cells were seeded in 24-well plates and incubated at 37 °C, 5% CO_2_ overnight. To identify T lymphocyte populations, cells were stained with the following mouse anti-human antibodies: anti-CD45 BUV395, anti-CD3 APC-R700, anti-CD4 BUV737, anti-CD8 BV785, anti-CD25 BV421, anti-CD127 BB700, anti-CD183 PE, anti-CD194 BV510, anti-CD196 BV650 (all from BD Biosciences, Milan, Italy) and anti-Nicotinic Acetylcholine Receptor α7/CHRNA7 Alexa Fluor 488 (Santa Cruz Biotechnology, Dallas, TX, USA). After 20 min incubation at 4 °C, cells were washed and resuspended in PBS, then acquired on a LSR Fortessa X-20 flow cytometer (Becton Dickinson, Milan, Italy). Data were analyzed using FACS Diva software v8.0.2. Treg, Th1, Th2, and Th17 lymphocytes were identified by the expression of the following combinations of markers on CD4^+^ T lymphocytes: CD25^hi^ CD127^−^ (Treg), CD183^+^ (Th1), CD194^+^ CD196^−^ (Th2), and CD194^+^ CD196^+^ (Th17) [30]. Gating strategy used to identify the specific T-cell subsets (Treg, Th1, Th2, and Th17) is reported as Supplementary Figure S1. The supplementary figure illustrates the sequential gating approach adopted for the identification and characterization of each T-cell population and provides additional methodological details to facilitate reproducibility and interpretation of the immunophenotyping results.

### 2.8. Virus Detection

TTV load was assessed in PBMCs, where the highest viral load has been reported [31,32]. TTV loads were expressed as the number of viral DNA copies per μg of genomic DNA extracted from PBMCs. The lower limit of detection was 10 copies of TTV per μg genomic DNA. Reference values in healthy cohorts are typically 2.3 ± 0.7 or 2.8 ± 1.09 log_10_ copies/mL [32,33], and a plasma TTV load of approximately 4 log_10_ copies/mL has been proposed as a practical threshold indicating altered immune function [34]. Due to the small sample size, patients were divided into two groups: (1) TTV viremia <4 log_10_ copies/mL, and (2) TTV viremia ≥4 log_10_ copies/mL. All other viruses, including influenza A (H1N1), human polyomaviruses (BKPyV and JCPyV), and herpesviruses (Varicella-Zoster virus, VZV), were detected as previously described [35].

### 2.9. Statistical Methods

Descriptive statistics were reported as percentages for categorical variables and as means ± standard deviations (SD) for continuous variables. Group differences were assessed using the Student’s *t*-test or the Mann–Whitney U test, as appropriate according to the distribution of the variables.

Survival analyses were performed using Cox proportional hazards regression models to assess the association between variables of interest and all-cause mortality. The proportional hazards assumption was evaluated by testing the correlation between Schoenfeld residuals and survival time. When the proportional hazards assumption was violated, survival distributions were compared using the Breslow (Wilcoxon) test.

Given the limited sample size and the relatively low number of events, the number of covariates included in multivariable models was intentionally restricted to minimize the risk of model over-parameterization and unstable estimates. Consequently, potentially relevant confounding factors, including Global Initiative for Chronic Obstructive Lung Disease (GOLD) stage, forced expiratory volume in one second (FEV1), and comorbidities, could not be comprehensively incorporated into the final models. Therefore, the observed associations should be considered exploratory and hypothesis-generating.

To assess the overall robustness and goodness-of-fit of the survival models, global model performance was evaluated using the Likelihood Ratio Test (LRT), comparing each fitted Cox model with the corresponding null model. In addition, the events-per-variable (EPV) ratio was considered to evaluate model stability and the potential risk of overfitting.

As a sensitivity analysis, Firth’s penalized Cox regression was performed to reduce small-sample bias and assess the robustness of hazard ratio estimates under conditions of limited sample size and sparse data.

No formal a priori sample size or power calculation was performed because this was an exploratory observational study based on an available clinical cohort. Accordingly, the study was not specifically powered to detect small-to-moderate effect sizes, and findings should be interpreted with appropriate caution.

Statistical significance was defined as a two-sided *p*-value ≤ 0.05. All statistical analyses were performed using SPSS software (version 26.0; IBM Corp., Armonk, NY, USA). Firth’s penalized Cox regression was performed using R Package: coxphf 1.13.4.

## 3. Results

This study cohort included 102 participants, comprising 38 current smokers and 64 never/former smokers.

Demographic characteristics such as sex (54.9% female overall; *p* = NS), age (72.64 ± 8.76 years; *p* = NS), body mass index (BMI, 27.27 ± 7.88 kg/m^2^; *p* = NS), and vegetable consumption (59.7%; *p* = NS) did not differ significantly between smoking groups.

Regarding nicotinic receptors, no statistically significant association was observed between smoking status and the distribution of the Chr15q25 genetic variant (rs16969968) (*p* = 0.096). Nevertheless, given the relatively small sample size and the proximity of the *p*-value to the conventional significance threshold, a potential relationship cannot be excluded and should be evaluated in larger studies. Among current smokers, 18.8% carried the G/G wild-type genotype, 12.5% the A/A genotype, and 68.8% the A/G genotype, whereas among never/former smokers the corresponding frequencies were 27.7%, 27.7%, and 44.7%, respectively.

Differences in α7nAChR expression in CD4^+^ cells were observed: current smokers had lower mean fluorescence intensity (MFI) than never/former smokers (35.46 ± 41.34 vs. 61.25 ± 37.61; *p* = 0.050, borderline) but a higher percentage of α7nAChR^+^ CD4^+^ cells (65.61 ± 21.23% vs. 46.50 ± 21.66%; *p* = 0.030).

Oxidative stress parameters were significantly higher in current smokers. Tail intensity (%) and 8-OHdG levels were elevated in smokers compared with never/former smokers (22.69 ± 6.01 vs. 16.83 ± 7.05, *p* = 0.001; 32.64 ± 13.17 vs. 22.01 ± 10.04 pg/mL, *p* = 0.002, respectively). MDA and IL-6 did not differ significantly between groups (*p* = NS). Total bilirubin was lower in current smokers (0.64 ± 0.32 mg/dL) compared with never/former smokers (0.84 ± 0.49 mg/dL; *p* = 0.031).

Among hematological parameters, current smokers exhibited significantly higher hemoglobin levels (13.55 ± 1.79 vs. 12.19 ± 1.71 g/dL, *p* = 0.004), total white blood cell counts (11.19 ± 5.28 vs. 9.43 ± 2.81 × 10^3^/μL, *p* = 0.031), and neutrophil counts (8.5 ± 4.7 vs. 6.9 ± 2.6 × 10^3^/μL, *p* = 0.031) compared with never/former smokers. In contrast, eosinophil counts did not differ significantly between the two groups (0.09 ± 0.12 vs. 0.12 ± 0.16 × 10^3^/μL, *p* = 0.412).

Immunological parameters also showed differences: the proportion of CD4^+^ T cells was higher in current smokers (72.18 ± 11.62% vs. 58.36 ± 19.59%, *p* = 0.028), while other lymphocyte subsets (CD8^+^, CD4/CD8 ratio, Treg, Th1, Th2, Th17, and Th17/Treg ratios) were not significantly different.

Among virological parameters, total TTV load differed significantly according to smoking status (*p* = 0.002), with a higher proportion of smokers having TTV >4 log_10_ copies/mL compared with never/former smokers (81.6% vs. 51.6%). Other viral markers, including BKPyV, JCPyV, and VZV, did not show significant differences between groups. All results are summarized in Table 1.

### Survival Analysis

Survival analyses were performed to evaluate the association between smoking-related biomarkers, immune parameters, and five-year mortality (Table 2).

In the primary multivariable Cox proportional hazards model including cotinine (COT), Torque teno virus (TTV ≥4 log copies/mL), total bilirubin, white blood cell count (WBC), and FEV1 (n = 34; 34 events), WBC emerged as an independent predictor of mortality. Each 1-unit increase in WBC was associated with a 12% higher risk of death (HR 1.12, 95% CI 1.01–1.24, *p* = 0.030). Total bilirubin showed a borderline protective association (HR 0.37, 95% CI 0.13–1.06, *p* = 0.064), whereas smoking status, TTV load, and FEV1 were not significantly associated with survival. Model discrimination was moderate (C-index = 0.65).

Given the limited sample size and events-per-variable ratio (EPV ≈ 6.8), a sensitivity analysis was performed using Firth’s penalized Cox regression. The penalized model yielded highly comparable results, with WBC remaining significantly associated with mortality (HR 1.12, 95% CI 1.01–1.23, *p* = 0.035) and total bilirubin maintaining a borderline protective effect (HR 0.39, 95% CI 0.13–1.01, *p* = 0.054). Neither smoking status (HR 1.03, 95% CI 0.43–2.95, *p* = 0.946), TTV load (HR 0.85, 95% CI 0.35–2.04, *p* = 0.710), nor FEV1 (HR 1.005, 95% CI 0.987–1.022, *p* = 0.566) showed significant associations with mortality.

To increase statistical power, a second multivariable model excluding TTV was subsequently fitted in the larger cohort (n = 50; 50 events). In this analysis, total bilirubin demonstrated a significant protective association with mortality (HR 0.46, 95% CI 0.22–0.97, *p* = 0.041), whereas WBC showed a borderline association with increased risk (HR 1.08, 95% CI 1.00–1.17, *p* = 0.065). Smoking status and FEV1 were not significantly associated with survival. Model discrimination remained comparable (C-index = 0.63).

Sensitivity analysis using Firth’s penalized Cox regression confirmed the stability of these findings. Total bilirubin remained significantly associated with reduced mortality risk (HR 0.47, 95% CI 0.22–0.95, *p* = 0.035), whereas the association between WBC and mortality remained borderline (HR 1.08, 95% CI 0.99–1.17, *p* = 0.067). Smoking status (HR 0.90, 95% CI 0.47–1.82, *p* = 0.752) and FEV1 (HR 1.006, 95% CI 0.992–1.020, *p* = 0.422) were not associated with mortality.

Assessment of global model performance using the Likelihood Ratio Test (LRT) indicated that neither multivariable model achieved overall statistical significance. In the first model, the LRT yielded χ^2^ = 7.36 with 5 degrees of freedom (*p* = 0.195), whereas in the second model the LRT yielded χ^2^ = 6.99 with 4 degrees of freedom (*p* = 0.136). The stronger evidence observed in the second model was consistent with its larger sample size and more favorable EPV ratio (approximately 12.5 versus 6.8 in the first model). The wide confidence intervals and non-significant global tests suggest limited statistical power to detect small-to-moderate effects.

Overall, elevated WBC counts were consistently associated with increased mortality risk, while higher total bilirubin levels showed a reproducible protective association. In contrast, smoking status, TTV load, and FEV1 were not independently associated with five-year survival. The concordance between standard and Firth-penalized Cox models supports the robustness of these findings despite the limited sample size.

## 4. Discussion

In this study, we investigated the interplay between smoking exposure, genetic susceptibility, oxidative stress, immune dysregulation, virological markers, and long-term outcomes in patients with severe-to-very-severe COPD undergoing pulmonary rehabilitation. Building on our previous findings of increased genomic damage in smoking COPD patients [35], we identified a distinct biological profile among current smokers characterized by a higher prevalence of the Chr15q25 rs16969968 risk genotype, increased oxidative DNA damage, altered hematological parameters, enhanced systemic inflammation, immune remodeling, and higher Torque teno virus (TTV) load. Collectively, these findings support the concept that smoking contributes to COPD progression through interconnected mechanisms involving genomic instability, oxidative stress, impaired immune regulation, and altered host–virus interactions.

The Chr15q25 rs16969968 polymorphism was more prevalent among current smokers than among never/former smokers, consistent with previous evidence linking this variant to nicotine dependence, smoking intensity, COPD susceptibility, and lung cancer risk [14,36]. This locus encodes a subunit of the nicotinic acetylcholine receptor complex and may influence smoking behavior through altered nicotine signaling. The observation that two current smokers carrying the AA genotype subsequently died from lung cancer further supports the hypothesis of a gene–environment interaction amplifying susceptibility to smoking-related pulmonary disease. Although the sample size precludes definitive conclusions, these findings are consistent with large population studies demonstrating that genetic predisposition may modulate the biological consequences of chronic tobacco exposure [14].

Hematological analyses revealed significantly higher hemoglobin concentrations, total leukocyte counts, and neutrophil counts in smokers. Elevated hemoglobin levels in the absence of increased erythrocyte counts are compatible with secondary polycythemia related to chronic carbon monoxide exposure and tissue hypoxia [37]. This adaptation may contribute to pulmonary hypertension, right ventricular strain, and thromboembolic complications frequently observed in advanced COPD [38]. Interestingly, the mean hemoglobin concentration observed among smokers approached values previously associated with increased mortality risk in large epidemiological studies [39].

Smoking was associated with marked evidence of oxidative stress and impaired antioxidant defenses. Current smokers exhibited significantly higher DNA damage, as reflected by increased tail intensity and 8-OHdG levels, together with lower circulating bilirubin concentrations. Cigarette smoke contains large amounts of reactive oxygen and nitrogen species capable of inducing DNA strand breaks, oxidative base modifications, lipid peroxidation, and mitochondrial dysfunction [40,41]. Among the biomarkers assessed, 8-OHdG is widely recognized as a sensitive indicator of oxidative DNA damage and has been associated with cardiovascular disease, cancer development, and adverse respiratory outcomes [16,42,43]. Conversely, bilirubin exerts potent antioxidant and cytoprotective effects through scavenging of reactive oxygen species and inhibition of NADPH oxidase activity [44,45]. Previous studies and meta-analyses have reported positive associations between bilirubin levels, lung function preservation, and survival in COPD [46]. Our findings extend this evidence by demonstrating a simultaneous increase in oxidative damage and reduction in endogenous antioxidant capacity among smokers with advanced disease.

Recent evidence suggests that oxidative stress may also contribute to the release of circulating nuclear and mitochondrial DNA fragments. Cigarette smoke induces cellular injury, apoptosis, and necrosis, resulting in the liberation of cell-free nuclear DNA (cf-nDNA) and mitochondrial DNA (cf-mtDNA) into the circulation. Emerging studies have shown that circulating cf-mtDNA and cf-nDNA levels are associated with COPD severity, exacerbation frequency, and mortality risk, suggesting that these biomarkers may integrate multiple dimensions of disease pathophysiology, including tissue injury, inflammation, and oxidative stress [47,48]. When combined with clinical and functional measurements, circulating cell-free DNA may provide additional insights into disease progression and prognosis. Although these biomarkers were not assessed in the present study, our findings support the broader concept that genomic instability and DNA damage represent central components of COPD pathogenesis and potential targets for future precision medicine approaches.

The increase in leukocyte and neutrophil counts further supports the presence of chronic systemic inflammation. Neutrophils represent key effector cells in COPD pathogenesis through the release of proteases, reactive oxygen species, inflammatory mediators, and neutrophil extracellular traps (NETs), all of which contribute to tissue destruction and airway remodeling [19,20]. Experimental evidence indicates that nicotine itself can directly stimulate NET formation [49], potentially linking smoking exposure to sustained inflammatory activation. Consistent with this hypothesis, WBC count emerged as the most robust prognostic marker in our survival analyses, suggesting that persistent systemic inflammation may be a major determinant of adverse outcomes in advanced COPD.

The immune alterations observed in smokers extended beyond innate immunity. Current smokers exhibited significantly higher percentages of CD4^+^ T lymphocytes, whereas CD8^+^ T cells, Treg cells, and Th1, Th2, and Th17 subsets did not differ significantly between groups. The increase in CD4^+^ cells in the absence of a proportional expansion of regulatory T cells may indicate a relative imbalance between immune activation and immune suppression. Such a pattern is compatible with a persistent pro-inflammatory state that may contribute to chronic tissue injury and impaired immune homeostasis.

Particularly intriguing were the findings related to α7 nicotinic acetylcholine receptor (α7nAChR) expression. Smokers showed a higher proportion of α7nAChR-positive CD4^+^ T cells despite lower receptor mean fluorescence intensity. Although apparently paradoxical, this pattern may reflect receptor remodeling induced by chronic nicotine exposure. One possibility is that prolonged stimulation increases the proportion of cells expressing detectable receptor levels while simultaneously reducing receptor density per cell. Alternatively, chronic nicotine exposure may induce receptor desensitization, altered receptor trafficking, or compensatory downregulation. Because receptor signaling and downstream functional responses were not assessed, these interpretations remain speculative. Nevertheless, the findings suggest that cholinergic signaling pathways may undergo substantial remodeling in smokers with advanced COPD and deserve further mechanistic investigation.

A novel aspect of our study was the assessment of TTV load as a surrogate marker of immune competence and susceptibility to persistent viral replication. Smokers exhibited significantly higher TTV loads than never/former smokers, with more than 80% of smokers showing viral loads above 4 log_10_ copies/mL. TTV is increasingly recognized as a dynamic marker of immune surveillance, as elevated viral replication generally reflects reduced immunological containment. The absence of significant differences in the prevalence of BKPyV, JCPyV, CMV, and VZV suggests that smoking may selectively influence the balance between host immunity and persistent viral replication rather than simply increasing the prevalence of latent viral infections.

The coexistence of elevated TTV load and increased oxidative DNA damage is particularly noteworthy. Although our study was not designed to establish causal relationships between these phenomena, accumulating evidence suggests that viral replication and host DNA damage responses are closely interconnected. Viruses can induce DNA damage and exploit DNA repair pathways to facilitate replication [50,51,52]. Recent observations have shown that individuals with high TTV viremia exhibit increased genomic instability, providing the first direct evidence linking TTV replication to DNA damage responses [53]. Since TTV is a single-stranded DNA virus that depends on host replication machinery, elevated viral replication may reflect broader alterations in genomic maintenance mechanisms. In COPD, where defective DNA repair pathways have already been described [54], smoking-induced oxidative stress may therefore contribute to DNA damage both directly and indirectly through impaired immune control of persistent viral replication. Rather than representing independent processes, oxidative DNA damage and elevated TTV load may constitute interconnected manifestations of smoking-induced biological dysregulation.

The prognostic analyses further highlighted the clinical importance of systemic inflammation and antioxidant defenses. In the primary multivariable model, WBC count emerged as an independent predictor of five-year mortality, whereas total bilirubin showed a protective trend. Because the number of events was limited, we performed sensitivity analyses using Firth’s penalized Cox regression, a method specifically designed to reduce small-sample bias and improve estimate stability in low events-per-variable settings. The penalized models produced highly consistent results, confirming the association between elevated WBC counts and increased mortality risk while preserving the protective effect of bilirubin. In the larger model, bilirubin remained independently associated with improved survival, whereas WBC retained a borderline association with mortality. Importantly, smoking status, TTV load, and FEV1 were not independently associated with mortality in either conventional or penalized analyses. The inclusion of FEV1 did not materially alter the estimates of the other covariates, suggesting that the prognostic effects observed were not merely reflections of airflow limitation severity.

These findings should be interpreted in light of the overall performance of the survival models. Neither model achieved global statistical significance in likelihood ratio testing, and confidence intervals remained relatively wide for several variables. Together with the exploratory nature of the study and the absence of a formal a priori power calculation, these findings indicate that the study may have been underpowered to detect small-to-moderate associations. Nevertheless, the consistency of the results across standard and penalized models supports the biological relevance of the observed associations and reduces concerns regarding model overfitting.

Dietary factors may also influence oxidative stress and genomic stability. Vegetable consumption was included as a proxy indicator of antioxidant intake because vegetables provide vitamins, carotenoids, polyphenols, and other bioactive compounds capable of modulating oxidative damage [55]. Human intervention studies using the Comet assay have shown that diets rich in vegetables and plant-derived foods are associated with reduced DNA damage and improved genomic stability [56], while recent meta-analyses have reported inverse associations between fruit and vegetable consumption and lung cancer risk, particularly among smokers [57]. Importantly, vegetable consumption did not differ significantly between smoking groups in our cohort, indicating that the higher levels of oxidative DNA damage observed among smokers are unlikely to be explained by dietary differences alone. However, residual confounding related to unmeasured dietary factors cannot be completely excluded.

### 4.1. Interpretation of Negative Findings and Prognostic Implications

An important aspect of the present study is the interpretation of several negative findings. Despite significant differences in TTV load between smokers and never/former smokers, TTV did not independently predict five-year mortality after multivariable adjustment. This observation suggests that TTV may be more useful as a marker of immune competence and viral control than as a direct prognostic biomarker. Indeed, elevated TTV replication may reflect underlying immune dysregulation without necessarily translating into increased mortality risk in patients with advanced COPD.

Similarly, smoking status itself was not independently associated with mortality. Although smoking is a well-established cause of COPD development and progression, its prognostic effect in cohorts with advanced disease may be mediated through downstream biological consequences, including systemic inflammation, oxidative stress, genomic instability, and immune dysfunction. In this context, biomarkers reflecting these biological processes, such as WBC count and bilirubin, may provide more direct prognostic information than smoking status alone.

Another notable finding was the lack of an independent association between FEV1 and survival. While airflow limitation remains central to COPD diagnosis and staging, increasing evidence indicates that mortality in advanced COPD is influenced by multiple extrapulmonary factors, including systemic inflammation, cardiovascular comorbidities, nutritional status, frailty, and immune dysfunction. The absence of a significant association between FEV1 and mortality in the present cohort may therefore reflect both the relatively homogeneous severity of airflow limitation among participants and the multifactorial nature of disease progression in advanced COPD.

Likewise, no significant differences were observed in several immune cell subsets, including CD8^+^ T cells, Treg cells, Th1, Th2, and Th17 populations. These negative findings suggest that smoking-related immune alterations in severe COPD may be selective rather than generalized, preferentially affecting specific immune compartments such as CD4^+^ T cells and cholinergic signaling pathways. Alternatively, chronic disease severity, pharmacological treatment, and individual variability may have obscured more subtle differences.

The absence of significant associations for BKPyV, JCPyV, CMV, and VZV prevalence also deserves consideration. These findings indicate that smoking may not uniformly increase susceptibility to all latent viral infections. Rather, smoking-related immune dysfunction may preferentially affect the control of persistent viral replication, as reflected by TTV load, without necessarily altering the prevalence of latent viral carriage.

Finally, neither multivariable survival model achieved overall statistical significance in likelihood ratio testing. Although this finding may partly reflect limited statistical power, it also highlights the complexity of COPD prognosis and suggests that additional biological and clinical determinants remain unmeasured. Consequently, the present findings should be viewed as exploratory and hypothesis-generating rather than definitive evidence of causal prognostic relationships.

### 4.2. Implications for Respiratory Rehabilitation

These multidimensional alterations have important implications for respiratory rehabilitation, which remains the most effective non-pharmacological intervention for COPD [58,59]. The strong associations between systemic inflammation, oxidative stress, immune imbalance, viral dysregulation, and clinical outcomes suggest that rehabilitation should be approached as a comprehensive therapeutic strategy, not just exercise training.

Elevated WBC and neutrophilia indicate persistent inflammation that may be modifiable through structured physical training and lifestyle interventions. The imbalance between oxidative stress and antioxidant defenses supports integrating nutritional counseling and antioxidant strategies within rehabilitation programs. The combination of increased CD4^+^ T cells, stable Tregs, and elevated TTV load suggests impaired immune regulation and antiviral control; pulmonary rehabilitation has been shown to improve immune surveillance and reduce infection risk [60]. Moreover, TTV monitoring alongside inflammatory and oxidative markers could aid patient stratification for rehabilitation intensity. Finally, genetic susceptibility and nicotine dependence reinforce the importance of incorporating structured smoking cessation within rehabilitation programs.

Overall, these findings support a precision-medicine approach to respiratory rehabilitation, tailoring interventions to inflammatory status, oxidative balance, immune competence, viral burden, and behavioral risk factors.

### 4.3. Strengths and Limitations

This study has several strengths. First, it adopted a multidimensional approach integrating genetic susceptibility, smoking exposure, oxidative stress biomarkers, genomic instability, immune phenotyping, virological markers, and long-term clinical outcomes within a well-characterized cohort of patients with severe-to-very-severe COPD. This comprehensive framework enabled the investigation of multiple interconnected biological pathways that are rarely assessed simultaneously in advanced COPD.

Second, the study combined established clinical and laboratory markers with emerging biomarkers, including TTV load as a surrogate indicator of immune competence and viral replication control, α7nAChR expression on T-cell subsets, and quantitative measures of oxidative DNA damage. The integration of these complementary biomarkers provides novel insights into the complex interactions between smoking, immune dysregulation, oxidative stress, and host resilience.

Third, the availability of five-year follow-up data allowed evaluation of the prognostic relevance of the investigated biomarkers. Furthermore, the use of sensitivity analyses based on Firth’s penalized Cox regression strengthened the reliability of the survival findings by addressing potential small-sample bias and reducing concerns regarding overfitting.

Fourth, the study was conducted in a clinically homogeneous population of patients with severe and very severe COPD undergoing pulmonary rehabilitation, thereby reducing disease heterogeneity and enabling a more focused assessment of smoking-related biological alterations.

However, several limitations should also be acknowledged. First, the relatively small sample size and limited number of events reduced statistical power and increased the risk of model overfitting, particularly in the primary survival model, where the events-per-variable ratio was below commonly recommended thresholds. Although sensitivity analyses using Firth’s penalized Cox regression yielded consistent results and supported the robustness of the main findings, the wide confidence intervals and non-significant likelihood ratio tests indicate that the study may have been underpowered to detect small-to-moderate associations.

Second, the observational and cross-sectional nature of most analyses precludes causal inference. Consequently, the relationships observed among smoking exposure, oxidative stress, immune alterations, viral replication, and mortality should be interpreted as associations rather than evidence of causality.

Third, despite adjustment for selected covariates, residual confounding cannot be excluded. Because of the limited sample size, clinically relevant variables such as GOLD stage, comorbidity burden, exacerbation history, nutritional status, and other potential prognostic factors could not be comprehensively incorporated into the multivariable models. Although FEV1 was included in the survival analyses, it was not independently associated with mortality and did not materially alter the observed associations; however, the study may not have been adequately powered to detect its prognostic contribution.

Fourth, smoking exposure was primarily assessed according to current smoking status and urinary cotinine levels, which objectively verified recent tobacco exposure. However, cumulative smoking burden, smoking intensity over time, passive smoking exposure, and longitudinal smoking trajectories were not comprehensively assessed. Moreover, the duration of smoking cessation among former smokers could not be accurately incorporated into the analyses and may have contributed to residual confounding. Therefore, the long-term biological effects of prior smoking exposure may not have been fully captured.

Fifth, although smokers exhibited significantly higher TTV loads, TTV was not independently associated with mortality after multivariable adjustment. Therefore, the present study supports the use of TTV as a marker of immune competence rather than as an established prognostic biomarker. Similarly, the observed coexistence of elevated TTV load and increased oxidative DNA damage does not establish a direct mechanistic relationship between viral replication, genomic instability, and survival outcomes.

Sixth, virological analyses were limited to selected latent and persistent viruses and did not include comprehensive characterization of the respiratory virome, airway microbiome, or viral reactivation dynamics over time.

Seventh, the biological interpretation of α7nAChR findings is constrained by the absence of functional studies. Receptor signaling activity, downstream immune responses, receptor internalization, and desensitization mechanisms were not evaluated. Therefore, the biological significance of the observed expression patterns remains speculative.

Eighth, dietary assessment was based on selected items from the European Health Interview Survey and served primarily as a proxy measure of antioxidant intake. Although no differences in vegetable consumption were observed between smoking groups, residual dietary confounding cannot be excluded.

Ninth, all participants were receiving inhaled corticosteroids together with methylcysteine-based therapy, which may have influenced inflammatory markers, immune cell phenotypes, oxidative stress biomarkers, and viral replication patterns.

Finally, the moderate discriminatory performance of the survival models, together with the absence of significant global model fit in likelihood ratio testing, suggests that relevant prognostic determinants were not fully captured. Future prospective multicenter studies integrating clinical characteristics, pulmonary function, imaging, longitudinal smoking exposure, immune phenotyping, oxidative stress biomarkers, circulating cell-free DNA, virological markers, and multi-omics approaches will be necessary to validate and expand these findings.

## 5. Conclusions

Overall, our findings support a multidimensional model of COPD in which smoking acts not only as a direct toxic exposure but also as a driver of interconnected processes involving genetic susceptibility, oxidative stress, immune dysregulation, impaired antiviral surveillance, and systemic inflammation. Patients with severe-to-very-severe COPD who continued smoking exhibited a distinct biological profile characterized by increased oxidative DNA damage, higher inflammatory cell counts, immune activation, reduced antioxidant defenses, and elevated TTV load.

Among the biomarkers investigated, elevated WBC counts emerged as the most consistent predictor of adverse long-term outcomes, while higher bilirubin levels showed a protective association, highlighting the prognostic relevance of systemic inflammation and antioxidant capacity. In contrast, although TTV load and DNA damage markers were associated with smoking-related biological alterations, their independent prognostic value could not be demonstrated in this cohort.

These findings emphasize the interplay between inflammatory, oxidative, immunological, and viral pathways in COPD progression and support the development of precision-guided rehabilitation strategies. Integrating biological profiling with smoking cessation, nutritional support, and infection prevention may improve risk stratification and optimize long-term outcomes in patients with advanced COPD.

## Figures and Tables

**Table 1:** Clinical, biological, and virological parameters by smoking status.

Variable	All (n = 102)	Current Smokers (n = 38)	Never + Former Smokers (n = 64)	*p*-Value
Sex: Female/Male	56 (54.9%)/46 (45.1%)	23 (60.5%)/15 (39.5%)	33 (51.6%)/31 (48.4%)	NS
Age (years)	72.64 ± 8.76	73.11 ± 8.94	72.22 ± 8.70	NS
BMI (kg/m^2^)	27.27 ± 7.88	26.36 ± 7.22	27.91 ± 8.35	NS
Vegetable intake (yes)	46 (59.7%)	23 (71.9%)	23 (51.1%)	NS
**Nicotinic Receptors**				
Chr15q25 (rs16969968): G/G/A/A/A/G	19 (24.1%)/17 (21.5%)/43 (54.4%)	6 (18.8%)/4 (12.5%)/22 (68.8%)	13 (27.7%)/13 (27.7%)/21 (44.7%)	0.096
α7nAChR expression in CD4^+^ cells (MFI)	53.46 ± 40.11	35.46 ± 41.34	61.25 ± 37.61	0.050
α7nAChR^+^ CD4^+^ cells (%)	54.37 ± 22.55	65.61 ± 21.23	46.50 ± 21.66	0.030 *
**Oxidative Parameters**				
Tail intensity (%)	19.18 ± 7.22	22.69 ± 6.01	16.83 ± 7.05	0.001 *
8-OHdG (pg/mL)	25.30 ± 12.04	32.64 ± 13.17	22.01 ± 10.04	0.002 *
MDA (μM)	40.90 ± 11.67	40.40 ± 11.47	41.18 ± 11.95	NS
IL-6 (pg/mL)	89.64 ± 118.03	111.8 ± 161.6	82.43 ± 101.6	0.440
Total bilirubin (mg/dL)	0.76 ± 0.44	0.64 ± 0.32	0.84 ± 0.49	0.031 *
**Hematological and Immunological Parameters**				
RBC (×10^6^/μL)	4.38 ± 0.70	4.51 ± 0.73	4.30 ± 0.67	NS
Hgb (g/dL)	12.67 ± 1.84	13.55 ± 1.79	12.19 ± 1.71	0.004 *
WBC (×10^3^/μL)	10.07 ± 3.97	11.19 ± 5.28	9.43 ± 2.81	0.031 *
Neutrophils (×10^3^/μL)	7.48 ± 3.6	8.5 ± 4.7	6.9 ± 2.6	0.031 *
Eosinophils (×10^3^/μL)	0.11 ± 0.14	0.09 ± 0.12	0.12 ± 0.16	NS
Lymphocytes (×10^3^/μL)	1.84 ± 1.48	1.78 ± 0.95	1.87 ± 1.71	NS
CD4^+^ (%)	64.96 ± 17.21	72.18 ± 11.62	58.36 ± 19.59	0.028 *
CD8^+^ (%)	25.19 ± 11.89	24.11 ± 9.25	25.81 ± 13.3	NS
CD4/CD8 ratio	3.73 ± 3.69	3.60 ± 1.90	3.82 ± 4.45	NS
Treg CD25ʰⁱ CD127^−^ (%)	0.67 ± 0.85	0.80 ± 0.81	0.58 ± 0.88	NS
Th1 (CD183^+^) (%)	3.66 ± 4.65	3.58 ± 6.05	3.74 ± 2.70	NS
Th2 (CD194^+^CD196^−^) (%)	9.83 ± 9.63	6.98 ± 9.85	11.65 ± 9.22	NS
Th17 (CD194^+^CD196^+^) (%)	20.47 ± 17.67	23.54 ± 14.6	18.63 ± 19.33	NS
Th17/Treg ratio	56.35 ± 52.10	49.20 ± 39.93	61.81 ± 60.42	NS
**Virological Parameters**				
TTV < 4 log_10_/≥4 log_10_ copies/mL	38 (37.3%)/64 (62.7%)	7 (18.4%)/31 (81.6%)	31 (48.4%)/33 (51.6%)	0.002 *
BKPyV (positive)	31 (34.8%)	11 (32.4%)	20 (36.4%)	NS
JCPyV (positive)	48 (53.3%)	20 (58.8%)	28 (50.0%)	NS
CMV (positive)	19 (20.0%)	5 (13.3%)	14 (24.1%)	NS
VZV (positive)	49 (55.1%)	17 (51.5%)	32 (57.1%)	NS

* Statistically significant (*p* ≤ 0.05). Abbreviations: BMI, body mass index; MFI, mean fluorescence intensity; MDA, malondialdehyde; NS, not significative; 8-OHdG, 8-hydroxy-2′-deoxyguanosine; IL-6, interleukin-6; RBC, red blood cells; Hgb, hemoglobin; WBC, white blood cell count; TTV, Torque teno virus; BKPyV, BK polyomavirus; JCPyV, JC polyomavirus; CMV, cytomegalovirus; VZV, varicella-zoster virus.

**Table 2:** Multivariable Cox proportional hazards regression and Firth-penalized Cox regression analyses for 5-year mortality.

Variable	Cox HR (95% CI)	*p*-Value	Firth HR (95% CI)	*p*-Value
**Model 1 (n = 34, 34 events)**				
Smoking status (Never/Former vs. Current)	1.09 (0.41–2.93)	0.863	1.03 (0.43–2.95)	0.946
TTV ≥ 4 log_10_ copies/mL	0.81 (0.33–1.99)	0.647	0.85 (0.35–2.04)	0.71
Total bilirubin	0.37 (0.13–1.06)	0.064	0.39 (0.13–1.01)	0.054
WBC (×10^3^/μL)	1.12 (1.01–1.24)	0.03	1.12 (1.01–1.23)	0.035
FEV1 (%)	1.005 (0.988–1.023)	0.576	1.005 (0.987–1.022)	0.566
**Model 2 (n = 50, 50 events)**				
Smoking status (Never/Former vs. Current)	0.92 (0.47–1.82)	0.819	0.90 (0.47–1.82)	0.752
Total bilirubin	0.46 (0.22–0.97)	0.041	0.47 (0.22–0.95)	0.035
WBC (×10^3^/μL)	1.08 (1.00–1.17)	0.065	1.08 (0.99–1.17)	0.067
FEV1 (%)	1.006 (0.992–1.020)	0.414	1.006 (0.992–1.020)	0.422

Abbreviations: HR, hazard ratio; CI, confidence interval; TTV, Torque teno virus; WBC, white blood cell count; FEV1, forced expiratory volume in one second. Model 1 included smoking status, TTV load, total bilirubin, WBC, and FEV1. Model 2 excluded TTV and was fitted in the larger cohort. Firth-penalized Cox regression was performed as a sensitivity analysis to reduce small-sample bias and assess the robustness of parameter estimates.

## Data Availability

The data that support the findings of this study are openly available in Zenodo at https://doi.org/10.5281/zenodo.11047757. Accessed on 27 January 2026.

## Supplementary Materials

The following supporting information can be downloaded at: https://www.mdpi.com/article/10.3390/biom16071009/s1. Table S1. Post-hoc pairwise comparisons of study biomarkers among never, former, and current smokers. Figure S1. Gating strategy used to identify the specific T-cell subsets (Treg, Th1, Th2, and Th17).

## References

[1] GBD 2019 Risk Factors Collaborators (2021). Global burden of 87 risk factors in 204 countries and territories, 1990–2019. Lancet.

[2] Vestbo J., Hurd S.S., Agustí A.G., Jones P.W., Vogelmeier C., Anzueto A., Barnes P.J., Fabbri L.M., Martinez F.J., Nishimura M. (2013). Global strategy for the diagnosis, management, and prevention of chronic obstructive pulmonary disease: GOLD executive summary. Am. J. Respir. Crit. Care Med..

[3] Agusti A., Calverley P.M., Celli B., Coxson H.O., Edwards L.D., Lomas D.A., MacNee W., Miller B.E., Rennard S., Silverman E.K. (2010). Characterisation of COPD heterogeneity in the ECLIPSE cohort. Respir. Res..

[4] Vanfleteren L.E., Spruit M.A., Groenen M., Gaffron S., van Empel V.P., Bruijnzeel P.L., Rutten E.P., Op ’t Roodt J., Wouters E.F., Franssen F.M. (2013). Clusters of comorbidities based on validated objective measurements and systemic inflammation in patients with chronic obstructive pulmonary disease. Am. J. Respir. Crit. Care Med..

[5] Santus P., Corsico A., Solidoro P., Braido F., Di Marco F., Scichilone N. (2014). Oxidative stress and respiratory system: Pharmacological and clinical reappraisal of N-acetylcysteine. COPD.

[6] Barnes P.J. (2020). Oxidative stress-based therapeutics in COPD. Redox Biol..

[7] Cho H.Y., Kleeberger S.R. (2020). Mitochondrial biology in airway pathogenesis and the role of NRF2. Arch. Pharm. Res..

[8] Ritchie A.I., Wedzicha J.A. (2022). Definition, causes, pathogenesis, and consequences of chronic obstructive pulmonary disease exacerbations. Eur. Respir. Rev..

[9] Drannik A.G., Pouladi M.A., Robbins C.S., Goncharova S.I., Bhatt D., Stämpfli M.R. (2004). Impact of cigarette smoke on clearance and inflammation after Pseudomonas aeruginosa infection. Am. J. Physiol. Lung Cell. Mol. Physiol..

[10] Gao M., Aveyard P., Lindson N., Hartmann-Boyce J., Watkinson P., Young D., Coupland C., Clift A.K., Harrison D., Gould D. (2022). Association between smoking, e-cigarette use and severe COVID-19: A cohort study. Int. J. Epidemiol..

[11] Pang X., Liu X. (2024). Immune Dysregulation in Chronic Obstructive Pulmonary Disease. Immunol. Investig..

[12] Stolz D., Mkorombindo T., Schumann D.M., Agusti A., Ash S.Y., Bafadhel M., Bai C., Chalmers J.D., Criner G.J., Dharmage S.C. (2022). Towards the elimination of chronic obstructive pulmonary disease: A Lancet Commission. Lancet.

[13] Agusti A., Melén E., DeMeo D.L., Breyer-Kohansal R., Faner R. (2022). Pathogenesis of chronic obstructive pulmonary disease: Understanding the contributions of gene–environment interactions across the lifespan. Lancet Respir. Med..

[14] Thorgeirsson T.E., Geller F., Sulem P., Rafnar T., Wiste A., Magnusson K.P., Manolescu A., Thorleifsson G., Stefansson H., Ingason A. (2008). A variant associated with nicotine dependence, lung cancer and peripheral arterial disease. Nature.

[15] McGuinness A.J., Sapey E. (2017). Oxidative Stress in COPD: Sources, Markers, and Potential Mechanisms. J. Clin. Med..

[16] Kasai H. (1997). Analysis of a form of oxidative DNA damage, 8-hydroxy-2′-deoxyguanosine, as a marker of cellular oxidative stress during carcinogenesis. Mutat. Res..

[17] Monroy-Iglesias M.J., Moss C., Beckmann K., Hammar N., Walldius G., Bosco C., Van Hemelrijck M., Santaolalla A. (2021). Serum Total Bilirubin and Risk of Cancer: A Swedish Cohort Study and Meta-Analysis. Cancers.

[18] Neofytou E., Tzortzaki E.G., Chatziantoniou A., Siafakas N.M. (2012). DNA Damage Due to Oxidative Stress in Chronic Obstructive Pulmonary Disease (COPD). Int. J. Mol. Sci..

[19] Liu L., Zhou L., Wang L.L., Zheng P.D., Zhang F.Q., Mao Z.Y., Zhang H.J., Liu H.G. (2023). Programmed Cell Death in Asthma: Apoptosis, Autophagy, Pyroptosis, Ferroptosis, and Necroptosis. J. Inflamm. Res..

[20] Zuo Y., Yalavarthi S., Shi H., Gockman K., Zuo M., Madison J.A., Blair C., Weber A., Barnes B.J., Egeblad M. (2020). Neutrophil extracellular traps in COVID-19. JCI Insight.

[21] Qin K., Xu B., Pang M., Wang H., Yu B. (2022). The functions of CD4 T-helper lymphocytes in chronic obstructive pulmonary disease. Acta Biochim. Biophys. Sin..

[22] Stolz D., Papakonstantinou E., Grize L., Schilter D., Strobel W., Louis R., Schindler C., Hirsch H.H., Tamm M. (2019). Time-course of upper respiratory tract viral infection and COPD exacerbation. Eur. Respir. J..

[23] Kulifaj D., Durgueil-Lariviere B., Meynier F., Munteanu E., Pichon N., Dubé M., Joannes M., Essig M., Hantz S., Barranger C. (2018). Development of a standardized real time PCR for Torque teno viruses (TTV) viral load detection and quantification: A new tool for immune monitoring. J. Clin. Virol..

[24] (2015). Quality Management Systems—Requirements.

[25] (2016). Medical Devices—Quality Management Systems—Requirements For regulatory Purposes.

[26] Directorate F: Social Statistics and Information Society Unit F-5: Health and Food Safety Statistics. EHIS Wave 1 Guidelines. https://ec.europa.eu/eurostat/documents/203647/203710/EHIS_wave_1_guidelines.pdf/ffbeb62c-8f64-4151-938c-9ef171d148e0.

[27] Miller V., Mente A., Dehghan M., Rangarajan S., Zhang X., Swaminathan S., Dagenais G., Gupta R., Mohan V., Lear S. (2017). Prospective Urban Rural Epidemiology (PURE) study investigators. Fruit, vegetable, and legume intake, and cardiovascular disease and deaths in 18 countries (PURE): A prospective cohort study. Lancet.

[28] Saber Cherif L., Diabasana Z., Perotin J.-M., Ancel J., Petit L.M.G., Devilliers M.A., Bonnomet A., Lalun N., Delepine G., Maskos U. (2022). The Nicotinic Receptor Polymorphism rs16969968 Is Associated with Airway Remodeling and Inflammatory Dysregulation in COPD Patients. Cells.

[29] Ilari S., Vitiello L., Russo P., Proietti S., Milić M., Muscoli C., Cardaci V., Tomino C., Bonassi G., Bonassi S. (2021). Daily Vegetables Intake and Response to COPD Rehabilitation. The Role of Oxidative Stress, Inflammation and DNA Damage. Nutrients.

[30] Romagnani S. (2000). T-cell subsets (Th1 versus Th2). Ann. Allergy Asthma Immunol..

[31] Focosi D., Antonelli G., Pistello M., Maggi F. (2016). Torquetenovirus: The human virome from bench to bedside. Clin. Microbiol. Infect..

[32] Maggi F., Pifferi M., Fornai C., Andreoli E., Tempestini E., Vatteroni M., Presciuttini S., Marchi S., Pietrobelli A., Boner A. (2003). TT virus in the nasal secretions of children with acute respiratory diseases: Relations to viremia and disease severity. J. Virol..

[33] Focosi D., Spezia P.G., Macera L., Salvadori S., Navarro D., Lanza M., Antonelli G., Pistello M., Maggi F. (2020). Assessment of prevalence and load of torquetenovirus viraemia in a large cohort of healthy blood donors. Clin. Microbiol. Infect..

[34] Mouton W., Conrad A., Bal A., Boccard M., Malcus C., Ducastelle-Lepretre S., Balsat M., Barraco F., Larcher M.V., Fossard G. (2020). Torque Teno Virus Viral Load as a Marker of Immune Function in Allogeneic Haematopoietic Stem Cell Transplantation Recipients. Viruses.

[35] Russo P., Milani F., De Iure A., Proietti S., Limongi D., Prezioso C., Checconi P., Zagà V., Novazzi F., Maggi F. (2024). Effect of Cigarette Smoking on Clinical and Molecular Endpoints in COPD Patients. Int. J. Mol. Sci..

[36] Thorgeirsson T.E., Gudbjartsson D.F., Surakka I., Vink J.M., Amin N., Geller F., Sulem P., Rafnar T., Esko T., Walter S. (2010). Sequence variants at CHRNB3–CHRNA6 and CYP2A6 affect smoking behavior. Nat. Genet..

[37] Nordenberg D., Yip R., Binkin N.J. (1990). The effect of cigarette smoking on hemoglobin levels and anemia screening. JAMA.

[38] Guidet B., Offenstadt G., Boffa G., Najman A., Baillou C., Hatzfeld C., Amstutz P. (1987). Polycythemia in chronic obstructive pulmonary disease. A study of serum and urine erythropoietin and medullary erythroid progenitors. Chest.

[39] Lee G., Choi S., Kim K., Yun J.M., Son J.S., Jeong S.M., Kim S.M., Park S.M. (2018). Association of Hemoglobin Concentration and Its Change With Cardiovascular and All-Cause Mortality. J. Am. Heart Assoc..

[40] Spencer J.P., Jenner A., Chimel K., Aruoma O.I., Cross C.E., Wu R., Halliwell B. (1995). DNA damage in human respiratory tract epithelial cells: Damage by gas phase cigarette smoke apparently involves attack by reactive nitrogen species in addition to oxygen radicals. FEBS Lett..

[41] Seo Y.S., Park J.M., Kim J.H., Lee M.Y. (2023). Cigarette Smoke-Induced Reactive Oxygen Species Formation: A Concise Review. Antioxidants.

[42] Foksinski M., Kotzbach R., Szymanski W., Olinski R. (2000). The level of typical biomarker of oxidative stress 8-hydroxy-2′-deoxyguanosine decreases with the advancing of the endometriosis. Free Radic. Biol. Med..

[43] Praticò D. (2008). Oxidative stress hypothesis in Alzheimer’s disease: A reappraisal. Trends Pharmacol. Sci..

[44] Mancuso C., Barone E. (2009). The heme oxygenase/biliverdin reductase pathway in drug research and development. Curr. Drug Metab..

[45] Stocker R., Yamamoto Y., McDonagh A.F., Glazer A.N., Ames B.N. (1987). Bilirubin is an antioxidant of possible physiological importance. Science.

[46] MacDonald D.M., Kunisaki K.M., Wilt T.J., Baldomero A.K. (2021). Serum bilirubin and chronic obstructive pulmonary disease (COPD): A systematic review. BMC Pulm. Med..

[47] Ware S.A., Kliment C.R., Giordano L., Redding K.M., Rumsey W.L., Bates S., Zhang Y., Sciurba F.C., Nouraie S.M., Kaufman B.A. (2024). Cell-free DNA levels associate with COPD exacerbations and mortality. Respir. Res..

[48] Giordano L., Ware S.A., Lagranha C.J., Kaufman B.A. (2025). Mitochondrial DNA signals driving immune responses: Why, How, Where?. Cell Commun. Signal..

[49] Hosseinzadeh A., Thompson P.R., Segal B.H., Urban C.F. (2016). Nicotine induces neutrophil extracellular traps. J. Leukoc. Biol..

[50] Li D., Lopez A., Sandoval C., Nichols Doyle R., Fregoso O.I. (2020). HIV Vpr Modulates the Host DNA Damage Response at Two Independent Steps to Damage DNA and Repress Double-Strand DNA Break Repair. mBio.

[51] Ryan E.L., Hollingworth R., Grand R.J. (2016). Activation of the DNA Damage Response by RNA Viruses. Biomolecules.

[52] Lopez A., Nichols Doyle R., Sandoval C., Nisson K., Yang V., Fregoso O.I. (2022). Viral Modulation of the DNA Damage Response and Innate Immunity: Two Sides of the Same Coin. J. Mol. Biol..

[53] Russo P., Milani F., Limongi D., Prezioso C., Novazzi F., Ferrante F.D., Maggi F., Antonelli G., Bonassi S. (2025). The effect of torque teno virus (TTV) infection on clinical outcomes, genomic integrity, and mortality in COPD patients. Mech. Ageing Dev..

[54] Sauler M., Lamontagne M., Finnemore E., Herazo-Maya J.D., Tedrow J., Zhang X., Morneau J.E., Sciurba F., Timens W., Paré P.D. (2018). The DNA repair transcriptome in severe COPD. Eur. Respir. J..

[55] Arshad Z., Shahid S., Hasnain A., Yaseen E., Rahimi M. (2025). Functional Foods Enriched With Bioactive Compounds: Therapeutic Potential and Technological Innovations. Food Sci. Nutr..

[56] Mišík M., Staudinger M., Kundi M., Worel N., Nersesyan A., Ferk F., Dusinska M., Azqueta A., Møller P., Knasmueller S. (2023). Use of the single cell gel electrophoresis assay for the detection of DNA-protective dietary factors: Results of human intervention studies. Mutat. Res. Rev. Mutat. Res..

[57] Tabatabaei S.V.A., Haghdoost A.A., Alavi S.M., Rajabzadeh-Dehkordi M., Ghalandari H., Askarpour M. (2025). Fruit and Vegetable Intake in Relation to Lung Cancer Risk: A Systematic Review and Dose-response Meta-analysis of Prospective Cohort Studies. J. Cancer Prev..

[58] McCarthy B., Casey D., Devane D., Murphy K., Murphy E., Lacasse Y. (2015). Pulmonary rehabilitation for chronic obstructive pulmonary disease. Cochrane Database Syst. Rev..

[59] Spruit M.A., Pitta F., Garvey C., ZuWallack R.L., Roberts C.M., Collins E.G., Goldstein R., McNamara R., Surpas P., Atsuyoshi K. (2014). Differences in content and organisational aspects of pulmonary rehabilitation programmes. Eur. Respir. J..

[60] Abbasi A., Wang D., Stringer W.W., Casaburi R., Rossiter H.B. (2024). Immune system benefits of pulmonary rehabilitation in chronic obstructive pulmonary disease. Exp. Physiol..

