# Supplementary material for: Effects of Cigarette Smoking on Oxidative Stress, DNA Damage, Immunological Profile, Viral Susceptibility, and Survival in Patients with Chronic Obstructive Pulmonary Disease
Source: Biomolecules. 2026 Jul 10;16(7):1009. doi: 10.3390/biom16071009 (PMC13406268; doi:10.3390/biom16071009)
Supplement: Supplementary file 1 [file biomolecules-16-01009-s001.zip › biomolecules-4372619-supplementary.pdf]

Table S1. Post-hoc pairwise comparisons of study biomarkers among never, former, and current smokers.

**Variable**	**Never vs Former Smokers**	**Former vs Current Smokers**	**Never vs Current Smokers**
Tail Intensity	0.952	0.001	0.034
8-OHdG	0.979	0.007	NS
CD4 count	0.359	0.032	NS
N_ASS_CD4	0.917	NS	NS
White blood cell count	0.930	NS	NS
TTV viral load	0.396	NS	0.042

Post-hoc pairwise comparisons among never smokers, former smokers, and current smokers. Data are reported as p-values. Statistically significant differences ($p < 0.05$) are shown in bold. No significant differences were observed between never smokers and former smokers for any of the investigated biomarkers, whereas current smokers differed significantly from one or both of the other groups. NS, not statistically significant ($p > 0.05$).

Figure S1. Gating strategy used to identify the specific T-cell subsets (Treg, Th1, Th2, and Th17).
