# Supplementary figures and images for: Effects of Cigarette Smoking on Oxidative Stress, DNA Damage, Immunological Profile, Viral Susceptibility, and Survival in Patients with Chronic Obstructive Pulmonary Disease
Source: Biomolecules. 2026 Jul 10;16(7):1009. doi: 10.3390/biom16071009 (PMC13406268; doi:10.3390/biom16071009)

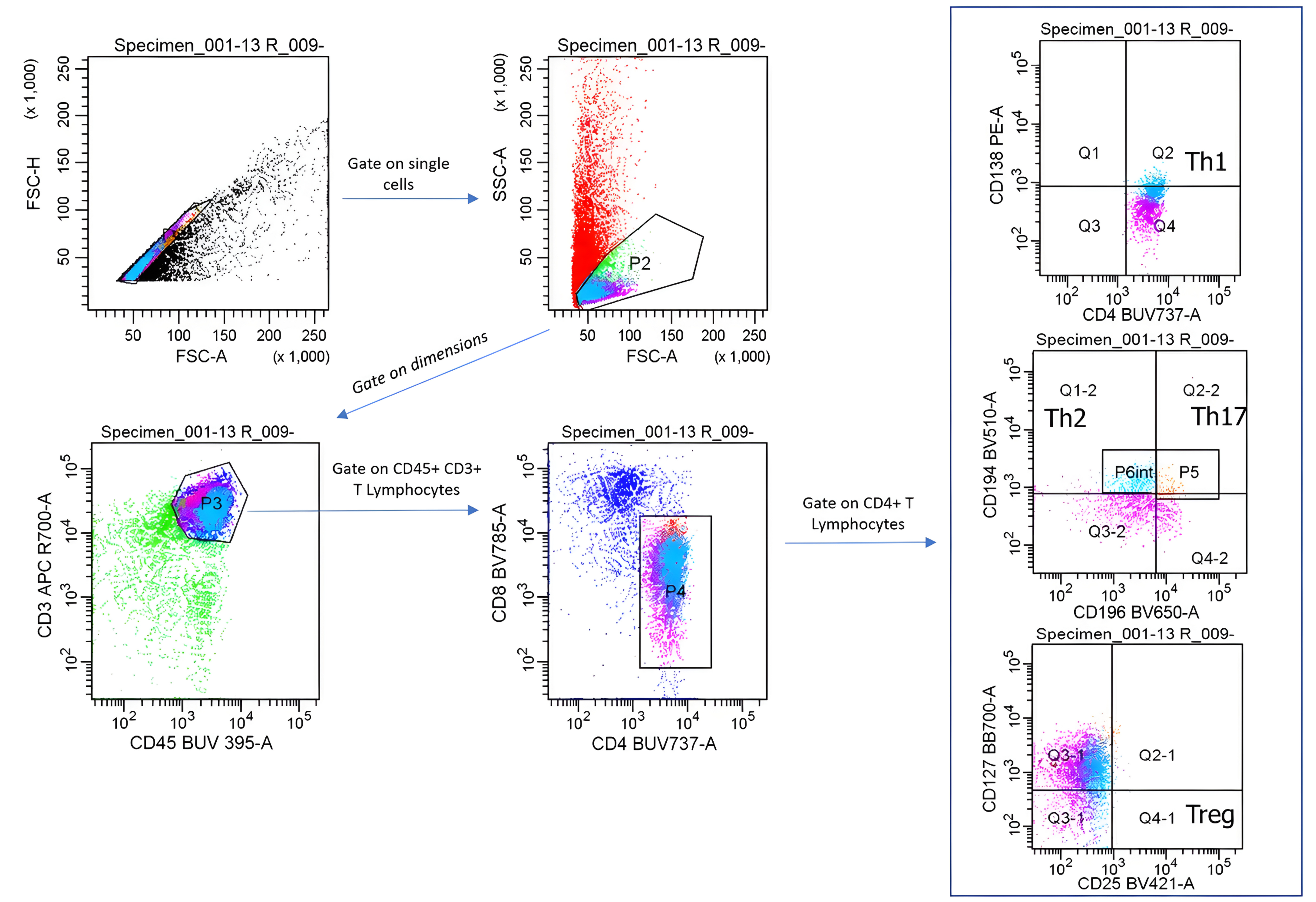

Supplement: Supplementary file 1 [file biomolecules-16-01009-s001.zip › biomolecules-4372619-Figure S1.JPG]
